# An embedded implementation research initiative to tackle service delivery bottlenecks in the expanded programme on immunisation in Pakistan: Overview and reflections

**DOI:** 10.7189/jogh.11.06003

**Published:** 2021-05-08

**Authors:** ASM Shahabuddin, Alyssa Sharkey, Faraz Khalid, Kumanan Rasanathan, Andreas Hasman, Nhan Tran, Ayesha Durrani, Kennedy Ongwae, Richard Duncan, Khawaja Aftab Ahmed, Saadia Farrukh, Paul Rutter, Debra Jackson, Assad Hafeez, Stefan Swartling Peterson, Abdul Ghaffar

**Affiliations:** 1 Implementation Research and Delivery Science Unit, Health Section, Programme Division, UNICEF, New York, USA; 2 Universal Health Coverage/Health Systems Department, WHO Regional Office for Eastern Mediterranean; 3 World Health Organization, Phnom Penh, Cambodia; 4 Regional Office for South Asia, UNICEF, Kathmandu, Nepal; 5 Alliance for Health Policy and Systems Research, World Health Organisation, Geneva, Switzerland; 6 UNICEF Country Office, Islamabad, Pakistan; 7 UNICEF Country Office, Sanaa, Yemen; 8 Immunisation Unit, Health Section, Programme Division, UNICEF, New York, USA; 9 London School of Hygiene and Tropical Medicine, London, UK; 10 School of Public Health, University of the Western Cape, Cape Town, South Africa; 11 Ministry of National Health Services, Regulations & Coordination, Islamabad, Pakistan; 12 Office of the Associate Director for Health, Programme Division, UNICEF, New York, USA

## Abstract

**Background:**

Embedded implementation research (IR) can play a critical role in health systems strengthening by tackling systems and implementation bottlenecks of a program. To achieve this aim, with the financial support of GAVI, the Vaccine Alliance, in 2016, the Government of Pakistan, UNICEF and the Alliance for Health Policy and Systems Research (AHPSR) launched an Embedded IR for Immunisation Initiative (the Initiative) to explore health systems and implementation bottlenecks, and potential strategies to tackle such bottlenecks in the Expanded Programme on Immunisation (EPI) in Pakistan. In total, 10 research teams were involved in the Initiative, which was the first of its kind in the country. In this paper, we provided a brief overview of the Initiative’s approach as well as the key learnings including challenges and successes of the research teams which could inform future embedded IR Initiatives.

**Methods:**

Data were collected from members of the IR teams through an online survey. In addition, in-depth interviews were conducted via phone and in-person from IR team members to explore further the challenges they faced while conducting IR in Pakistan and recommendations for future IR initiatives. The qualitative information obtained from these sources was collated and categorized into themes reflecting some of the challenges, successes, and lessons learned, as well as teams’ recommendations for future initiatives.

**Results:**

The embedded IR Initiative in Pakistan followed several steps starting with a desk review to compile information on key implementation challenges of EPI and ended with a dissemination workshop where all the research teams shared their IR results with policymakers and implementers. Key factors that facilitated the successful and timely completion of the studies included appreciation by and leadership of implementers in generation and use of local knowledge, identification of research priorities jointly by EPI managers and researchers and provision of continuous and high-quality support from in-country research partners. Participants in the Initiative indicated that challenges included a lack of clarity on the role and responsibilities of each partner involved and need for further support to facilitate use and dissemination of research findings.

**Conclusions:**

The Initiative established that an immunisation programme in a lower middle-income country can use small and time-bound embedded IR, based on partnerships between programme managers and local researchers, to generate information and evidence that can inform decision-making. Future embedded IR initiatives should strive to ensure effective coordination and active participation of all key stakeholders, a clear research utilisation plan from the outset, and efforts to strengthen research teams’ capacity to foster utilisation of research findings.

Embedded implementation research (IR) aims to make research relevant and applicable to decision-making [1-4] by integrating research methods and approaches within existing health programme implementation and policymaking cycles to improve service delivery and overcome bottlenecks [3-8]. By addressing research questions of direct relevance to programmes temporally aligned with implementation cycles and getting implementer and policy maker buy-in through direct engagement, embedded research aims to increase the likelihood of findings being applied and leading to actual system and service improvements – and in this way can contribute to progress towards Universal Health Coverage (UHC) [9].

In Pakistan only two-thirds of children receive all basic vaccinations [10], less than the global average of about 85 per cent [11,12]. The country is currently home to two million unvaccinated children and approximately half of all deaths among children under 5 years of age occur due to vaccine-preventable diseases such as diarrhoea, pneumonia, and meningococcal disease [10]. Despite substantial progress during the past two decades, the immunisation coverage in Pakistan remains low with persistent disparities within the country.

In this context, in 2016, the Government of Pakistan launched an Embedded Implementation Research for Immunisation Initiative (the Initiative) to explore health system bottlenecks and strategies to increase coverage and equity in the national immunisation programme. With the financial support of Gavi, the Vaccine Alliance, the Initiative was a collaborative effort by the Government of Pakistan, UNICEF and the Alliance for Health Policy and Systems Research (AHPSR). In addition, in-country technical support was provided by the Pakistan Health Services Academy (HSA) and an in-country consultant researcher contracted by UNICEF Pakistan.

The Initiative provided unique insights into specific implementation challenges and strategies to resolve them. In this paper, we summarise the Initiative’s approach, as well as reflections on challenges, successes and lessons learned in order to inform future embedded IR Initiatives.

## Approach

**Figure 1:**
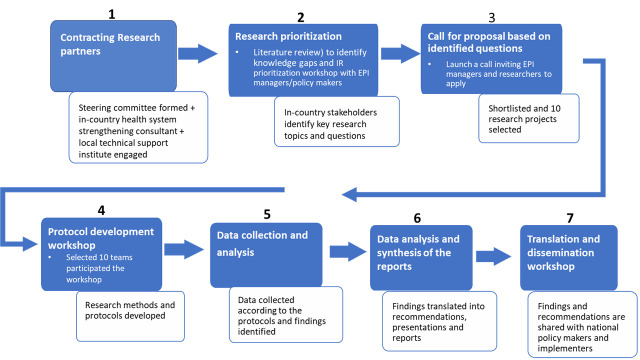
Steps and key milestones of the implementation research initiative.

The Initiative followed several steps, starting with a desk review to compile existing data and information on key implementation challenges (**Figure 1**). Following this, a research prioritisation workshop involving EPI managers and in-country researchers was organized in February 2017 to prioritize topics for research, and a call for proposals was subsequently made. From 36 submissions, 10 research teams were selected for funding (**Table 1**) based on predefined criteria independently assessed by a committee consisting of IR experts from UNICEF, AHPSR and other organisations. The criteria included: a research team comprised of EPI implementers (drawn from policy makers, managers and frontline workers) as principal investigators, and one or more in-country researchers as academic partners; a research question relevant to EPI implementation challenges identified as priorities at the initial workshop; appropriate study design and methods; expected findings actionable by intended audiences; capacity of the research team; and feasibility in terms of budget and timing. In addition, the review committee aimed to include a diverse set of research projects in the Initiative, focusing on a diverse set of implementation challenges across districts and provinces. Projects selected received US$20 000 each. All projects were completed (including ethical approval, data collection and analysis, and dissemination of results) within a nine-month time period.

**Table 1:** Implementation research thematic areas, research topics, and study sites

Thematic area	Research topic	Study sites (district(s)/ province)
**Community and demand**	1. Involvement of community health workers to improve immunisation coverage in hard-to-reach areas of Sukkhur district	Sukkur/ Sindh
	2. Addressing community barriers to immunisation in Rajanpur district	Rajanpur/Punjab
	3. Addressing EPI vaccination demand through mHealth in Quetta City, Balochistan: A feasibility study	Quetta/Balochistan
	4. Social mobilisation campaign to tackle immunisation hesitancy in Sargodha and Khushab districts in Pakistan	Sarghoda and Khushab/Punjab
**Immunisation supply chain and performance management systems**	5. Visibility and Analytics Network (VAN) approach to improve immunisation supply chain and management performance system in Pakistan	Ghotki and Sukkur/Sindh
	6. E-Vaccs: Qualitative assessment of the barriers and enablers in implementation using Consolidated Framework for Implementation Research	Lahore, DG Khan, Gujrat, Rawalpindi/Punjab
**Human resources and service delivery**	7. Exploring opportunities for strengthening supportive supervision: A case study of EPI services in Sindh	Hyderabad and Thatta/Sindh
	8. Understanding accountability for human resources in EPI, Balochistan: Perspective from the government officials at the provincial and district levels	Pishin, Harnai, Jhal Magsi, Killa Abdullah, Killa Saifullah/Balochistan
**Building on the polio experience**	9. Examining the mechanisms and effectiveness of multi-tiered, EPI-polio synergy	Four provinces (Balochistan, Khyber Pakhtunkhwa, Punjab, and Sindh)
	10. Developing three-dimensional narrative to counter polio vaccine refusal in Charsadda	Charsadda/ Khyber Pakhtunkhwa

## METHODS USED TO GATHER REFLECTIONS

In addition to the insights gleaned on specific implementation challenges faced by the EPI in Pakistan and potential strategies to resolve them, the Initiative afforded an opportunity for those involved to reflect on challenges, successes and key learnings from the approach.

In addition to review of key documents relating to the Initiative, we conducted an online survey among self-selected members of the research teams (n = 13), asking about experiences and outcomes (**Table 2**). The survey consisted of open-ended questions regarding participants’ experiences with the Initiative, such as which challenges were encountered, what were successful strategies, facilitating factors, and key lessons learned. In total, 13 respondents (10 researchers, 3 EPI managers), representing nine of the 10 IR teams participated. Additional face-to-face qualitative and open-ended interviews were conducted with other members of the teams. These activities were all carried out in English as members of the research teams were fluent. Thematic analysis was carried out to identify major themes. Two researchers carried out the analysis to rectify any inconsistencies in findings.

**Table 2:** Data collection methods and participants in survey and interviews*

Data collection methods	Survey and interview participants
Online survey	Researchers (n = 10)
	EPI implementers(n = 3)
In-depth interviews (face-to-face)	Researchers (n = 5)
	EPI implementers (n = 2)
	UNICEF staff (n = 2)
	UNICEF recruited local consultant researcher (n = 1)
	Representative of AHPSR (n = 1)
	EPI director (n = 1)

EPI – Expanded Programme on Immunisation

*EPI implementers included policy makers, managers and frontline health workers.

In addition to the survey and interviews, key supporting documents were analysed. Content from these sources was independently analysed by two researchers and categorized into themes relating to challenges, successes and key lessons learned. Before the survey, informed consent was obtained from all respondents through e-mail. In addition, verbal informed consent was obtained prior to the interview.

## RESULTS

### Appreciation by and leadership of implementers in generation and use of local knowledge

From the start, the Initiative was strongly supported by the Pakistan Ministry of National Health Services, Regulation and Coordination (MoNHSRC), which worked to engage all relevant stakeholders and facilitated cooperation between research teams and EPI authorities. One research team member commented:

*“*The overall concept and execution of this initiative was fully supported by the MoNHSRC. This was the key facilitating factor which encouraged all EPI managers and offices for their active participation into the Initiative*.” – EPI implementer (manager)*

EPI implementers engaged in research reported that they appreciated the Initiative and the opportunity to prioritise key implementation challenges in collaboration with their colleagues. The prioritised challenges informed the call for research proposals around a set of overarching themes (**Table 1**). These implementers also indicated that they appreciated the opportunity to work directly with researchers to identity challenges and solutions to strengthen the EPI in Pakistan. Local researchers within the research teams also expressed gratitude for the opportunity to work directly with EPI implementers and appreciated that the research had direct relevance to programme priorities.

The importance of designating EPI implementers as principal investigators for each project was also highlighted. There were several benefits to this approach noted by research team members, including that this facilitated introduction of the research to communities, data collection, and implementers’ ownership of the research. This approach also helped ensure the research topics were relevant, and that the focus of findings and recommendations was on practical and actionable solutions.

“This is one of the easiest data collection exercises of my 25 years of research career – the local health official in our team ensured that we are able to reach the lady health workers and traditional birth attendants (community health workers) in remotest areas of rural [a province].” *– Researcher*

### Identification of research priorities jointly by EPI managers and researchers

An early success was that the Initiative began with an inclusive consultative process with key stakeholders to specify and prioritize topics for implementation research.

As one team member stated:

“It was first of its type of research in which the implementors were engaged from the very beginning which helped to identify the real implementation issues and their solutions.” *– EPI implementer (manager)*

These overarching themes (relating to suboptimal community mobilisation and caregiver demand, inadequate supply chains, underperformance in human resources and service delivery, and lost opportunities to build on experiences from polio eradication) were translated into clear research questions and reflected in the 10 projects by researcher-implementer teams (**Table 1**).

“I always thought that research is for academics…being involved in this study right from identifying a relevant research question to synthesizing findings for policy makers, I am finding the research useful for me too….” *– EPI implementer (Vaccinator)*

### Provision of continuous and high-quality support by in-country researcher partners

When reflecting on the Initiative overall, the research teams reported that the approach (ie, literature review, orientation workshop, call for proposals, finalisation of study designs, data collection and analysis, and dissemination of findings and recommendations at a final workshop) was robust and enabled them to generate new evidence within a brief period. All teams reported that the ongoing support they received from the full-time, in-country consultant was critical throughout and helped to ensure timely completion of the work. The research consultant was a national of Pakistan with a deep understanding of the social and political context and who was experienced in both implementation and health systems research. This type of support was important for the ‘researchers’ within the teams as well, as many lacked expertise in the small, time-bound embedded implementation research approach.

“In my opinion three key successes were: 1) guidance and continuous technical support from the consultant researcher remained an integral component to complete all the projects within the allocated time; 2) involvement of EPI managers/implementers in the research; 3) addressing specific implementation barriers through each single research project.” *– Researcher*

Moreover, some research teams mentioned that the role of local research partners was limited, and that they contributed very little to technical oversight of the Initiative, including review of protocols, finalisation of research methodology, tools, data collection monitoring, and synthesis of final reports. This was a missed opportunity highlighted by a few respondents to develop the capacity of a local research/academic institution which could be used as an IR platform for liaising with federal and provincial governments, EPI, and other research/academic institutions in the country even after the end of the Initiative.

### Need of further attention in embedded implementation research to facilitate use and dissemination of research findings

It became apparent with some of the studies that including implementers as principal investigators of the research process was not sufficient to ensure full use of the research findings. The ten research teams (including both EPI managers and local researchers) had different skill sets, particularly relating to implementation research. A few teams struggled to present their research findings and recommendations in an effective manner to relevant policy-makers due to limited capacity in research dissemination and policy communication. Another major challenge related to limited capacity and skills on the strategies to ensure uptake of research to inform policy and practice or utilisation of research. The process to ensure use of IR recommendations in programmes and policies requires time and commitment to follow-up. Despite the involvement of implementers in the research, no team designated a focal person accountable for the translation of research recommendations into policy and practice. Furthermore, dissemination of findings and recommendations was primarily through a national-level dissemination workshop chaired by the Federal Ministry for Health. However, in light of the fact that Pakistan is a decentralized country, research teams stressed the need to organize more sub-national dissemination activities (ie, roundtables) to share clear and specific recommendations with provincial- and district-level EPI policy makers, particularly as some of their recommendations were more applicable to programming at these levels. This is particularly important to facilitate utilisation of the research findings in similar settings beyond that where the research was undertaken.

“Dissemination should be held in each province and districts and soft as well as hard copies of the clear recommendations should be shared with [relevant stakeholders].” *– EPI implementer (manager)*

“I would perhaps think of a more localized approach: 1) organizing roundtables in provinces to discuss key findings 2) reporting positive and negative in mainstream media and also publishing in peer-reviewed articles” *– Researcher*

The feedback we received iterated that careful consideration is necessary to identify the appropriate entry points for translation of IR findings and recommendations into policy and practice. Several research team members stated that they would have benefitted from additional technical and financial support to foster the use and application of relevant research findings following the completion of data analysis and reporting. In addition, feedback called for better participation of and coordination among all relevant district-, provincial- and federal-level stakeholders and officials, as well as engagement and support of relevant community groups.

“Research uptake would have been easier if we had active participation of all the stakeholders and officials at all levels. In addition, for some solutions political will and community participation would be cardinal, supreme and crucial.” *– Researcher*

### Need of clarity on the roles of each partner

Although the partners involved in the Initiative were aware of their roles and responsibilities, research teams expressed a lack of clarity on the various roles of these partners. For example, a few research team members mentioned that they were unclear about who to contact for logistical or technical support at various times throughout the Initiative. In addition, a few research team members indicated that there was a limited coordination among the partners and insufficient technical support from the local research partners.

“The project went smoothly because of [the] direct involvement of EPI managers and [the] in-country research consultant, however…there is a big hollow space in effective coordination and utilisation of research findings which needs to be filled through concrete and dedicated efforts by all stakeholders.”* – Researcher*

As the teams executed their studies, a few had internal disagreements about the distribution of workload and financials. In addition, funds disbursement to a few teams became an issue and posed delays in project execution. These respondents recommended that from the outset of the project, not only partners’ role and responsibilities should be clear, but also the distribution of incentives, either monetary or visibility/credit sharing. This would ensure that partners complement each other’s roles rather than compete.

## DISCUSSION

The experiences shared by research team members and partner agencies of the Pakistan Embedded Implementation Research for Immunisation Initiative can inform future embedded IR initiatives. With strong government buy-in, leadership and involvement, joint identification of research priorities by implementers and researchers, and ongoing technical support, the Initiative generated programme-relevant information in a short timeframe and with a relatively small budget. Challenges encountered by the research teams related to some confusion about the specific roles and responsibilities of the involved partners (particularly following the protocol development workshop), limited capacity on implementation research of some research teams and difficulties translating research findings and recommendations into practice by some teams.

This experience revealed how vital support and appreciation by the highest authorities within a system is to ensure the acceptance by and involvement of relevant stakeholders, and the successful implementation of a program. In Pakistan, leadership of the Federal Ministry of Health was instrumental to acceptance of the Initiative by all stakeholders.

The gap between researchers and policy makers or implementers has been a long-standing concern [13,14]. Implementers’ perspective is rarely taken into consideration in research, although they are the same individuals responsible for addressing service delivery bottlenecks. For several of the EPI managers engaged in this Initiative, it was the first time they were given the opportunity to identity research questions as well as to lead the research to explore solutions. Evidence from other settings suggests that involvement of implementers/policy-makers in research ensures greater relevance and uptake of research findings and recommendations into policy and programmatic decisions [3,4,15-21]. Additional evidence suggests that stakeholders’ ownership of the research is increased when they are involved in projects from the outset or lead the process [4,5,8].

However, the involvement of implementers in research, as in the embedded implementation research approach is necessary but not sufficient to ensure application of research findings within the immediate setting of the research or translation to other similar settings. Use of the research findings for EPI programmes and policies produced by this Initiative was challenging for some of the teams. This was mainly because of the lack of skills relating to research uptake among research teams, lack of planning on appropriate communication channels and dissemination products and not having a dedicated person or coordination body to ensure that findings are taken into consideration by the appropriate authorities. In some settings, this difficulty reflects insufficient decision-making power by the implementer involved in the research.

Effective research utilisation can be complex, and often depends on the context in which the research takes place, as well as the mandate, power, knowledge and skills of relevant stakeholders, including researchers and policy makers [16,20,22-24]. We found this to be true in this experience. Moreover, in a decentralized health system such as Pakistan’s, it is important that communication, and dissemination of discrete and specific recommendations should be done with policy makers at the appropriate levels at which decisions can be made. Engagement of communities to explore their roles in implementing new solutions can also be critical.

Building researchers, policy makers’ and programme managers’ capacity to foster research utilisation, working with strong advocates or policy champions, and having the support of a coordination body in the country have been found to improve uptake of IR findings and recommendations into EPI policies and practices [15,22,25,26]. Moreover, establishing an implementation research platform within the Ministry of Health supported from other stakeholders (eg, research institutions) may help both to build IR capacity in the country and to promote evidence-based health policy and practice. The feedback we received from research team members included that they faced some challenges due to lack of clarity on roles and responsibilities of all the involved partners throughout. Other analyses have similarly reported that effective coordination is often challenging, especially in settings where involvement of every partner is key [15,27]. Future embedded IR initiatives should be careful to identify and develop an implementation strategy (including a clear delineation of roles and responsibilities, as well as dissemination and communication of recommendations) prior to the start of research. Moreover, there should be a dedicated strategy or guideline which ensure effective coordination among all partners involved and ensure that each of the partners do their job as planned.

## CONCLUSIONS

The lessons presented here represent reflections shared by EPI implementers, researchers and agency partners who were involved in the Embedded IR for Immunisation Initiative in Pakistan. These lessons may guide future embedded IR initiatives in other settings, particularly if there is clarity on the roles and responsibilities of each shareholder outlined from the outset, effective coordination amongst the different partners, and efforts to strengthen research teams’ capacity to foster utilisation of research findings, when appropriate.
